# Role of Adiponectin in the Pathogenesis of Rheumatoid Arthritis

**DOI:** 10.3390/ijms21218265

**Published:** 2020-11-04

**Authors:** Kamila Szumilas, Paweł Szumilas, Sylwia Słuczanowska-Głąbowska, Katarzyna Zgutka, Andrzej Pawlik

**Affiliations:** 1 Department of Physiology, Pomeranian Medical University in Szczecin, 70-111 Szczecin, Poland; kamila.szumilas@pum.edu.pl (K.S.); sylwia@pum.edu.pl (S.S.-G.); katgrymula@o2.pl (K.Z.); 2 Department of Social Medicine and Public Health, Pomeranian Medical University, 71-210 Szczecin, Poland; pawszu@pum.edu.pl

**Keywords:** rheumatoid arthritis, adiponectin, adiponectin isoforms, pro-inflammation

## Abstract

Rheumatoid arthritis (RA) is a systemic chronic inflammatory autoimmune joint disease, characterized by progressive articular damage and joint dysfunction. One of the symptoms of this disease is persistent inflammatory infiltration of the synovial membrane, the principle site of inflammation in RA. In the affected conditions, the cells of the synovial membrane, fibroblast-like synoviocytes and macrophage-like synovial cells, produce enzymes degrading cartilage and underlining bone tissue, as well as cytokines increasing the infiltration of immune cells. In patients with RA, higher levels of adiponectin are measured in the serum and synovial fluid. Adiponectin, a secretory product that is mainly white adipose tissue, is a multifunctional protein with dual anti-inflammatory and pro-inflammatory properties. Several studies underline the fact that adiponectin can play an important pro-inflammatory role in the pathophysiology of RA via stimulating the secretion of inflammatory mediators. This narrative review is devoted to the presentation of recent knowledge on the role played by one of the adipokines produced by adipose tissue—adiponectin—in the pathogenesis of rheumatoid arthritis.

## 1. Introduction

**Figure 1:**
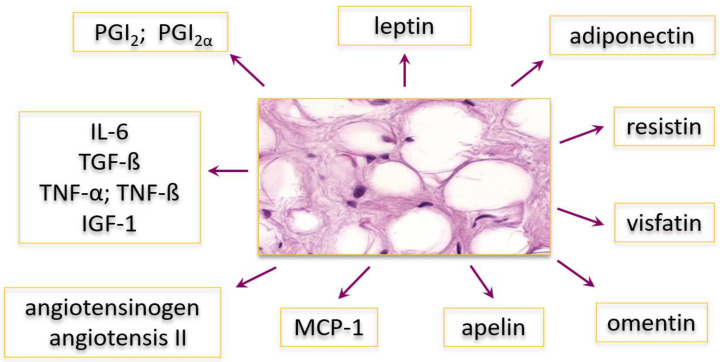
The main adipokines synthesized and secreted by adipocytes of white adipose tissue. Various type of adipokines are presented, including hormones, cytokines, and other biologically active substances: PGI—prostaglandins I2 and I2alpha; IL-6—interleukin 6; TGF-ß—transforming growth factor beta; TNF-α and TNF-ß—tumor necrosis factor alpha and beta; IGF-1—insulin-like growth factor 1; MCP-1—monocyte chemoattractant protein-1.

Adiponectin is a secretory product of adipocytes, cells with a very metabolically active white adipose tissue. The tissue secretes a number of biologically active substances, such as various cytokines, enzymes, and peptide hormones called adipokines (Figure 1), which play an important role in both physiological and pathological processes [1]. Among others, the secretory products of adipocytes may participate in modulating immune and inflammatory responses [2]. For this reason, overweight and obese people are more likely to develop pathological changes. Cytokines produced by excess fat tissue can affect the body’s natural processes, contributing to the development of inflammation. Inflammation is the reaction of vascularized connective tissue to damaging factors. The basis of many diseases is chronic inflammation that lasts for many years or even the rest of the patient’s life. Disrupted by inflammatory factors, physiological processes create a “vicious pathophysiological circle”, which the body is unable to break. Successive and cyclically repeated processes of tissue destruction and repair lead to organ dysfunction.

The aim of this review is to present recent knowledge on the role played by one of the adipokines produced by adipose tissue—adiponectin—in the pathogenesis of rheumatoid arthritis.

## 2. Rheumatoid Arthritis (RA)

Rheumatoid arthritis (RA), a systemic chronic inflammatory joint disease, develops in genetically susceptible individuals, not only under the influence of environmental factors, but also through epigenetic mechanisms. It is a heterogeneous disorder, with different pathogenetic mechanisms and various clinical forms [3,4,5]. RA is a severe autoimmune disease characterized by chronic symmetrical polysynovitis (arthritis) of the peripheral joints, leading to the destruction of cartilage and underlying bone tissue. This disease affects 1% of the world’s population and is associated with a loss of physical fitness, quality of life, and frequent comorbidities [5,6].

The structure ensuring the proper function of each joint is the synovial membrane and synovial fluid, enabling collision-free joint mobility. The synovial membrane is composed of loose proper connective tissue and some compacted connective tissue and yellow adipose tissue in places. It creates microplicae and microvilli that penetrate the joint cavity. In addition to the typical connective tissue cells and variable leukocyte populations, there are also two types of specialized cells: macrophage-like synovial cells (type A), which are derived from blood monocytes and remove tissue fragments from the synovial fluid by friction movements; and type B cells, which are fibroblast-like synoviocytes (FLS) that produce hyaluronan and small amounts of proteoglycans and form part of the synovial fluid. The synovium is rich in numerous blood vessels and contains a high density of fenestrated capillaries, in normal conditions situated very close to the synovial surface (an arrangement disrupted in rheumatoid synovium) with fenestrations preferentially oriented mainly toward the joint cavity [7].

Due to the action of an unidentified stimulus (bacteria, virus, and antigen), inflammation can develop in the synovium. This leads to vasodilation and increased permeability, followed by leukocyte infiltration and increased fibroblast proliferation. Activated rheumatoid arthritis synovial fibroblasts (RASFs) are characterized by an autonomic pathogenic phenotype that includes the ability to hyperproliferate and migrate, thus contributing to synovial hyperplasia and the spread of RA to healthy joints [3,7,8,9]. The ability of the cells to migration from affected to healthy joints was previously confirmed via in vitro and animal studies [9].

Likewise, resident and arriving monocytes, differentiating into RA synovial macrophages, show a pro-inflammatory profile that is associated with pathological RASF activity; the number of these cells in the synovium of affected joints correlates with disease activity and joint erosion [10,11,12]. In addition, it is widely accepted that endothelial cells in synovial inflammation also contribute to chronic synovitis through both angiogenesis and immune cell recruitment [5,13]. In this context, it is recognized that synovial fibroblasts and synovial macrophages play a key role in the course of RA pathology [3,5,8,14].

The disease is also characterized by the presence of immune cells and the production of autoantibodies such as anti-citrullinated protein antibodies (ACPA) and rheumatoid factor (RF), as well as increased levels, among others, of TNF-α, IL-1β, and IL-6, which are all believed to be central pro-inflammatory cytokines that participate in chronic synovitis, osteoclast formation, and subsequent erosive joint damage [3,5,8,14,15].

The process responsible for the synthesis of autoantibodies is under the control of follicular helper cells (Tfh), a specialized subset of CD4+ T lymphocytes, cooperating with B lymphocytes. Therefore, it is suggested that B cells, together with Tfh, play one of the important roles in the pathogenesis of RA [16,17].

Understanding the mechanisms underlying both immune regulation and reducing inflammatory conditions is critical to designing new treatment strategies for RA. One approach to understanding these mechanisms is to study the balance of the neuro–endocrine–immune system, which is crucial for maintaining homeostasis and effective adaptation to stressors [18,19]. The complex communications and their regulatory mechanisms are based on the presence of common mediators such as neurotransmitters, cytokines, hormones, and their receptors. In this aspect, on the one hand, dysregulation in the immune–endocrine integrated circuits is involved in the development of chronic metabolic diseases, including obesity, diabetes, and metabolic syndrome [20]. On the other hand, RA has been defined as an example of a disease resulting from abnormal interactions between these systems [21].

## 3. Adiponectin (ADP)

Human adiponectin, encoded by the *ADIPOQ* gene, is a secretory product, mainly of white adipocytes, and one of either bioactive peptides or proteins, immune molecules, or inflammatory mediators, with different biological activities. The expression of adiponectin was also identified in osteoblasts, liver parenchyma cells, myocytes, endothelial cells, and placenta [22]. Together with other members of the adipokine family, adiponectin plays a critical role in several major disorders, including insulin sensitivity, cardiovascular disease, conditions of arthritis, and obesity [23]. Since the discovery of the first adipokine, leptin, in 1994, profile studies have identified hundreds of adipokines in the human fat proteome (adipokinome), all of which can strongly modulate inflammation via the autocrine/paracrine and endocrine pathways [23].

Adiponectin was identified by several researchers, using different techniques, and is also known as Acrp30, adipoQ, ApM1, and GBP28; it is a 28–30 kDa secretory protein of adipocytes (Obata et al. 2013) and structurally consists of a fibrous subunit at the nitrogen terminus and a globular subunit at the carboxyl terminus [24]. It belongs to the soluble collagen superfamily and is homologous to complement factor C1q and the tumor necrosis factor (TNF) family [25].

The main biological functions of adiponectin include the stimulation of fatty acid biosynthesis and the inhibition of gluconeogenesis in the liver [25,26]. In addition, it is unclear whether glucose uptake in skeletal muscles can be applied through signaling pathways. Studies have shown that adiponectin can be used to improve insulin resistance by reducing the amount of intracellular fat through increased fatty acid oxidation via activating peroxisome proliferator-activated receptor alpha (PPARα) and enhancing insulin receptor substrate (IRS) signaling in skeletal muscle and liver [27,28]. Moreover, adiponectin was found to have antioxidant, anti-inflammatory, and anti-atherosclerotic effects [26,29].

Three isoforms of adiponectin are distinguished, depending on the degree of oligomerization as follows: low molecular weight (LMW) in the form a trimer of three adiponectin monomers, medium molecular weight (MMW) hexamer, and high molecular weight (HMW) multimer containing 12-32 adiponectin monomers [1,27,30].

Within isoforms, MMW hexamers and HMW multimers constitute the richest of the adiponectin circulating in the blood, while monomers are present in human plasma at very low levels LMW, and are normally not detected in the circulation [26,31]. Moreover, the serum also contains globular adiponectin (gAPN), a product of the proteolytic cleavage of the fibrous adiponectin (fAPN), probably with independent biological activities [1]. Adiponectin exerts its biological effects via binding with two main receptors; adiponectin receptor 1 (AdipoR1) and adiponectin receptor 2 (AdipoR2); these have a specific distribution and characteristics. AdipoR1 was identified for the first time in skeletal muscle, the other AdipoR2 in liver. Adiponectin receptor 1 is involved in modulation of AMP kinase, while AdipoR2 in activation of PPARα. However, it is unknown whether adiponectin receptors are expressed in multiple tissues. Both of them serve as receptors for globular and full-length adiponectin and mediate the increased fatty-acid oxidation and glucose [32].

Many data indicate that various of adiponectin isoforms are involved in the pathogenesis of various metabolic diseases such as type 2 diabetes, metabolic syndrome and related complications, especially cardiovascular diseases, and also autoimmune disease such as RA, systemic lupus erythematosus and osteoarthritis [33]. These isoforms have also been shown to act as acute phase reagents that influence inflammatory mechanisms in both the acute and chronic disease. The disruption of adiponectin isoform formation is one of the main availabilities in metabolic disorders [29]. Individual isoforms of adiponectin appear to affect gene expression differently [34].

Moreover, adiponectin isoforms differently influence the expression of lipid genes in primary human hepatocytes (PHH) [35]. Population studies have shown that HMW adiponectin negatively correlates with low-density lipoprotein cholesterol, triglycerides, apolipoprotein B, and apolipoprotein E, and is positively associated with high-density lipoprotein cholesterol [36,37,38]. In obesity, the formation of adiponectin isoforms is disrupted, which leads to the development of pathological conditions [39]. Given their pathophysiological effects, harmful adiponectin isoforms may possibly be a target of a therapeutic strategy while maintaining the beneficial effects of other adiponectin isoforms [40]. The high-molecular-weight (HMW) isoform of adiponectin is believed to be the most physiologically important and is increasingly used as a marker of disease-related adipocyte dysfunction [1,30,41,42].

Studies over the years revealed the dual functions of adiponectin, which have since been the subject of conflict between various researchers. It is known that the existing adiponectin duality in functions, e.g., anti-inflammatory and pro-inflammatory effects, contributes to the pathogenesis of many diseases [43,44,45,46]. The adiponectin exerts beneficial anti-inflammatory properties on the cardiovascular system, including atherosclerosis and metabolic disorders, such as obesity and insulin resistance. For example, in the vascular, the protective effects of HMW adiponectin are involved. The pro-inflammatory effect of adiponectin is observed in RA, chronic kidney disease (CKD), and inflammatory bowel disease, a group of intestinal disorders. A lack of understanding of these phenomena can be recognized as difficulties in the precise measurement of adiponectin isoforms or the lack of a universal standard [26,39].

As mentioned above, adiponectin, or multifunctional adipokine, can present both anti-inflammatory and pro-inflammatory effects, although the biological function of this cytokine appears to be influenced by exposure time, concentration, and environmental indications such as other cytokines and the tissue microenvironment. The above controversial evidence that adiponectin can have a dual inflammatory effect defines the specific roles of adiponectin in RA [1]. The anti-inflammatory properties of adiponectin may be a major component of its beneficial effects on cardiovascular and metabolic disorders, including atherosclerosis and insulin resistance [47].

## 4. Adiponectin and Rheumatoid Arthritis

Unlike other diseases, systemic autoimmune and chronic inflammatory joint diseases are characterized by the increased production of adipokines, and high adiponectin levels in plasma and synovial fluid have been confirmed in RA patients, when comparing their levels with the plasma of healthy subjects [26,48,49]. The differences between RA and healthy controls were also observed in serum adiponectin isoform contents. The level of HMW was higher, while there were no differences in MMW content, whereas globular adiponectin (gAPN) content, like HMW, was much higher in RA patients [26,50]. Because of these dependencies, it was suggested that the differences between RA patients and healthy individuals with regard to the levels of HMW and globular adiponectin could be regarded as potential RA biomarkers to evaluate the early stages of disease progression [26,51,52]. To establish the effect of adiponectin isoforms on primary human synovial fibroblast gene expression in RA, Frommer et al. (2012) stimulated the cells with native adiponectin and its three various isoforms. The stronger activation of gene expression in RA synovial fibroblasts and the production of pro-inflammatory chemokines and cytokines was exerted by HMW/MMW-enriched and globular adiponectin; as a consequence, the influx of lymphocytes was observed. The LMW adiponectin isoform led to the minimal expression of chemokines and cytokines [34]. However, there are conflicted data on the role of adiponectin in pathophysiology and progression of RA. The controversies can be connected with the ability of adiponectin to form multimeric complexes with various biological activity, and with ability to affect the expression of genes in rheumatoid FLS and other type of effector cells [53]. Additionally, the adiponectin receptors bind the multimeric forms with different affinity. It was shown that AdipoR1 receptor binds the adiponectin trimer with higher affinity, while AdipoR2 shows a greater affinity to higher-order multimers MMW and HMW [54]. Studies by Kontny et al. (2012) indicated that human rheumatoid FLS responded in different manner after stimulation with three forms of adiponectin isoforms. Both HMW and MMW presented pro-inflammatory and pro-destructive effects, while a third isoform, LMW adiponectin, showed the low activity level but stimulated the cells to stronger answer to IL-1ß [55]. The role of LMW adiponectin in RA is not well investigated; nevertheless, studies performed by Li at al. (2015) with Chinese female patients indicated that the level of LMW in the serum of the patients can be associated more with disease activity of RA than both HMW and MMW adiponectin multimers [56].

Several studies underline that adiponectin can play an important pro-inflammatory role in the pathogenesis of RA, particularly in the joints, via stimulating the secretion of inflammatory mediators, among others, by activated synovial fibroblasts that expressed adiponectin receptors [9]. An in vitro study with cultured RASFs revealed that the RASFs respond to adiponectin by increasing pro-inflammatory factors, including prostaglandins E2, IL-6, IL-8, matrix metalloproteinases-1, and -13 (MMPs-1, -13). The stronger pro-inflammatory effect showed HMW isoforms of adiponectin as compared to LMW isoform in these cells [5,57]. Inconsistent effects exerted via various isoforms of adiponectin have been noted in cells other than synovial fibroblasts, e.g., monocytes. The effects of adiponectin isoforms were investigated in cultured primary monocytes that were isolated from six healthy individuals. The results obtained indicated that the HWM adiponectin isoform induced the interleukin-6 (IL-6) secretion, compared to constitutive secretion, while LMW adiponectin reduced IL-6 secretion and increased IL-10 secretion in lipopolysaccharide (LPS)-activated monocytes [58].

These results are especially important because IL-6 is regarded to be the most pleiotropic cytokine, playing a key role in the development of chronic inflammation associated with RA. The pleiotropic effects IL-6 in the synovium include chronic synovitis, the proliferation of FLS, angiogenesis, and cartilage degradation [59].

Within immune cells, T lymphocytes are also attributed to the pathogenesis of RA, although the exact role remains unclear. Data in the literature suggest that mainly CD4 T-helper (Th) lymphocytes are included in RA pathogenesis via cytokine and chemokine secretion [15]. Follicular helper T (Tfh) cells are specialized T cells subset support B cells and are essential for germinal center formation, affinity maturation, and the development of the highest affinity antibodies and memory B cells. The cells expressed CXC chemokine receptor 5 required for the migration into B-cell follicles in secondary lymphoid organs [60,61].

It was also demonstrated that human RA FLSs in culture stimulated with adiponectin promoted the generation of Tfh lymphocytes, mainly mediated by the secretion of IL-6 [17]. In the in vitro study, RA fibroblast-like synoviocytes (FLS) isolated from the synovial tissue of RA patients were used. The RA FLS were stimulated with adiponectin and then co-cultured with CD4 T cells obtained from healthy patients. Results from the study noted that Tfh cells expressed AdipoR1; however, adiponectin was not directly included in Tfh lymphocyte generation in vitro, but indirectly via IL-6 production by adiponectin-stimulated RA LFSs [17]. In the other in vivo part of the study, DBA/1J (CIA, collagen-induced arthritis) mice were used. The intra-articular injection of adiponectin aggravated synovial inflammation and increased frequency of Tfh cells in the joints of mice [17].

In another study, taking into consideration the pro-inflammatory effects of adiponectin, Choi and co-authors (2020) investigated the stimulatory effect of adiponectin on the production of IL-6, IL-8, prostaglandin E2 (PGE2), VEGF, and MMPs. The in vitro studies were carried out with FLS derived from patients with RA. Additionally, the correlation between adiponectin and VEGF or MMPs was investigated by measuring the levels of these three proteins in the joint fluid of RA patients. The obtained results showed, for the first time, that adiponectin was able to induce the production of vascular endothelial growth factor (VEGF) and MMPs, namely MMP-1 and MMP-13, which can lead to joint inflammation and destruction. Furthermore, the level of adiponectin in the joint fluid of RA patients, was positively correlated with the level of VEGF [26,62,63,64].

In RA, chondrocytes of joint cartilage and bone cells underlying bone tissue are also included. Human and murine chondrocytes express functional adipokine receptors [65]. The chondrocytes isolated from human normal articular cartilage, C28/I2, C20A4, TC28a2 human immortalized chondrocytes, and the murine chondrogenic cell lines, treated with adiponectin, showed an increase, in a dose-dependent manner, in the expression of mRNA and MMP-3 secretion, through AdipoR1 [51,65,66]. The adiponectin-stimulated human primary chondrocytes obtained from 14 RA patients and the murine ATDC-5 cell line secretion of vascular cell adhesion molecules-1 (VCAM-1), identify factors responsible for leukocyte and monocyte infiltration at inflamed joints [2]. As Scotece et al. (2012) described, in human chondrocytes, RA patients’ adiponectin also induced nitric oxide (NO), IL-6, MMP-3, MMP-9, monocyte chemoattractant protein (MCP)-1, and IL-8 and promoted inflammation by increasing TNF-α, IL-6, and IL-8 [57,67].

To assess the association between serum levels of adiponectin in RA and radiographic damage, Giles et al. (2011) performed studies including RA patients of both sexes. Results of the studies showed that higher adiponectin levels in the patient were associated with more joint destruction, and the effect of adiponectin can depend on gender, obesity, and pharmacotherapies of RA patients [68].

Adiponectin can also affect the function of primary human osteoblasts and osteoclasts. After stimulation, adiponectin can modulate the secretory function of osteoblasts in vitro—cells producing bone matrix—to decrease the ability of cells to mineralize the matrix; also, adiponectin concurrently increases the activity of osteoclasts to resorb bone tissue. On the other hand, adiponectin stimulates the expression of MMP-9 and iron-containing tartrate-resistant acid phosphatase (TRAP) in osteoblasts and also increases the secretion of IL-8 by the cells. The study of human rheumatoid arthritis bone tissue revealed that adiponectin can also inhibit osterix (Osx) expression, an osteoblast-specific transcription factor that is essential for bone formation, and stimulate osteoprotegerin mRNA expression, which disturbs the inhibition of osteoclast activity, thereby delaying bone formation [26,69]. The process of the activation of osteoclast function in RA patients can be mediated via the increase of osteopontin synthesis by adiponectin-stimulated synovial fibroblasts. Osteopontin in bone tissue is required for osteoclast recruitment and the initiation of bone erosion. The regulatory role of adiponectin and osteopontin expression in RA was confirmed via in vitro studies with primary RA synovial fibroblasts and with a collagen-induced arthritis mice model in vivo, in which the extent of bone erosion was observed [70]. As mentioned above, adiponectin levels in serum and synovium are changed in RA patients, and the adiponectin exerts its biological effects through the activation of adiponectin receptors. However, there is no evidence of a correlation between AD-related gene polymorphisms and RA. A study by Zhao et al. (2020) was devoted to exploring the association between adiponectin and single-nucleotide polymorphisms (SNPs) in its receptor gene with genetic susceptibility to RA in a Chinese population [71]. A total of 617 male and female patients with RA and 639 geographically and ethnicity matched healthy controls were enrolled in the study, and five D SNPs (rs266729, rs2241766, rs1063537, rs2082940, and rs1063539) and two ADR SNPs (rs7539542 and rs12342) were genotyped. The results showed that adiponectin and adiponectin receptor polymorphisms were probably not associated with the occurrence and genetic susceptibility to RA. The adiponectin gene polymorphisms have a minor impact on the stimulation of anti-cyclic citrullinated peptide in female patients with RA; also, the polymorphisms have no association with the plasma adiponectin level [71].

Other cell types, such as endothelial cells, and lymphocytes revealed a predominantly pro-inflammatory response to adiponectin [5,57].

**Figure 2:**
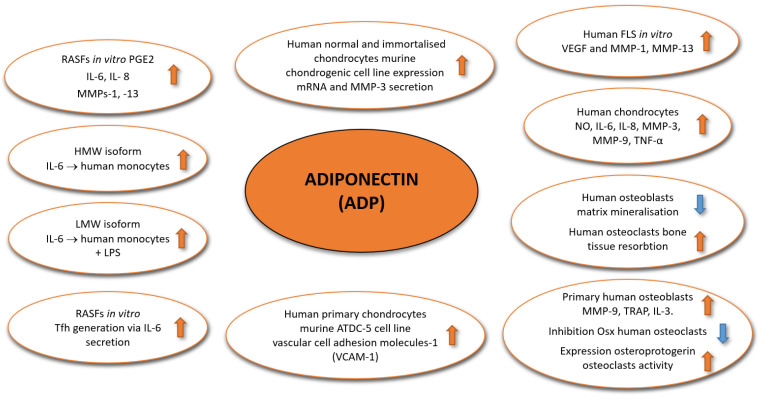
The effects of adiponectin and its isoforms on various cell types in RA.

All data clearly indicated that adiponectin, with a pro-inflammatory effect, is one of the main players in the development of RA (Figure 2 and Table 1).

## 5. Potential Therapeutics Targeting Adiponectin in RA

Adiponectin, multifunctional protein with pro-inflammatory properties, plays an important role in the development and progression of RA. The ability of adiponectin to multimerize and form three isoforms with different biological activity may be considered for use in the treatment of RA. The knowledge on functional activities of all adiponectin multimers can be permitted to block the isoforms to reduced RA progression and disease activity. The inhibition of individual adiponectin multimers, to reduce harmful adiponectin effects and promote its beneficial activity, is one hypothesis in RA therapy [34]. However, it can be taken into consideration that responses of target cells as RASF on adiponectin stimulation can differ among RA patients, and also to the different medication [76]. When looking for new possibilities in modulating adiponectin activity and therapy RA, adiponectin receptors and co-receptors should also be considered as a target [34,77,78,79]. The aim of the study performed by Lee et al. (2018) was to generate monoclonal antibodies against adiponectin isoforms as potential therapeutic agents in RA [40]. The experiment was performed in a mouse model, and monoclonal antibody (mAb) was obtained from sera of immunized BALB/c mice recognizing MMW adiponectin, and also mAbs recognizing three human isoforms of adiponectin from hybridoma cells. The human mAbs recognized all adiponectin isoforms in human serum and tissue [40]. Further exploration of the role of adiponectin in the pathophysiology of diseases, including RA, and its mechanisms of action may provide a tool for developing personalized therapeutic approaches based on modulating the activity of individual adiponectin multimers.

## 6. Adiponectin and Osteoarthritis

The activity of adiponectin is also noted in osteoarthritis (OA), a disease that is the most prevalent form of aging-related joint diseases, and which affects more than 37% of individuals aged over 60 years. In the etiology of OA, genetic and non-genetic factors are included, such as congenital defects, aging, gender, trauma, and obesity. Osteoarthritis may develop as a primary disease of the motor organ or as a secondary one in the course of other inflammatory joint diseases and is characterized by articular cartilage degradation, subchondral bone sclerosis, osteophyte formation, and synovial inflammation [80,81]. The meta-analysis presented in a review by Tang et al. (2018) showed that the systemic adiponectin concentration is higher in OA patients than in healthy controls and is also localized in the synovium [82]. Additionally, it was also shown that adiponectin has a pro-inflammatory effect in OA. Experimental in vitro studies with synovial fibroblasts revealed that adiponectin stimulates the production of key mediators of destructive arthritis, IL-6, and pro-MMP-1 [83]. Osteoarthritis is also characterized by osteophyte formation. In an investigation into the effects of adipokines on the development of OA osteophytes, adiponectin- and visfatin-stimulated osteoblasts, and chondrocytes, respectively, to increase their release of pro-inflammatory mediators [84]. After the treatment of cultured primary human chondrocytes from OA patients with adiponectin, the increased expression and secretion of MMP-1, MMP-3, and MMP-13 was observed [51,85,86].

## 7. Conclusions

Duality in the inflammatory functions show that adiponectin is one of the most widely investigated secretory products of white adipocytes. Data confirm the suggestion that the pro-inflammatory activity of adiponectin results in the development and/or increased severity of RA. Adiponectin affects the gene and protein expression in cells, not only in the synovial membrane, but also of chondrocytes, osteoblasts, osteoclasts, endothelial cells, and lymphocytes. The existence of adiponectin isoforms produces its multi-directional and complicated role in the signaling. Therefore, it is difficult to establish the most useful therapeutic strategy in inflammatory joint diseases.

## Figures and Tables

**Table 1:** Selected studies investigating the association of adiponectin and rheumatoid arthritis in humans, cell cultures, and animals.

Authors	Study Design	Subjects	Results/Outcomes
Su et al. 2015 [72]	Observation of the effect of adiponectin on the expression of oncostatin M (OSM), a pro-inflammatory cytokine, in human osteoblastic cells.	Human osteoblastic cells.	Adiponectin increased OSM expression via the PI3K, Akt, and NF-κB signaling pathways in osteoblastic cells, suggesting that adiponectin is a novel target for arthritis treatment.
Bustos Rivera-Bahena et al. (2015) [73]	Cross-sectional study evaluating the correlation between adipokine levels and disease activity.	121 RA patients	No correlation between serum adiponectin and clinical activity of RA, but a negative correlation with TNFα and positive correlation with IL-1β.
Chennareddy et al. (2016) [74]	Cross-sectional study evaluating the serum concentrations of adiponectin and its impact on disease activity and radiographic joint damage.	43 RA patients 25 controls	Increased levels of serum adiponectin in RA, but no correlation with erosive and non-erosive disease, disease duration, BMI, waist-to-hip ratio and disease activity.
Krumbholz et al. (2017) [69]	Adiponectin and its receptors were examined in bone tissue. Primary human osteoblasts and osteoclasts were stimulated with adiponectin and analyzed using real-time polymerase chain-reaction and immunoassays. Effects on matrix-production by osteoblasts and differentiation and resorptive activity of osteoclasts were examined.	Cell cultures	Adiponectin expression in key cells of bone remodeling. Adiponectin altered gene expression and cytokine release in osteoblasts and increased IL-8 secretion by osteoclasts. Adiponectin inhibited osterix and induced osteoprotegerin mRNA in osteoblasts. In osteoclasts, MMP-9 and tartrate resistant acid phosphatase expression was increased. Accordingly, the mineralization capacity of osteoblasts decreased, whereas the resorptive activity of osteoclasts increased.
Lee et al. (2018) [49]	Meta-analyses on serum/plasma adiponectin or visfatin levels in patients with RA and controls and the correlation coefficients between circulating adiponectin and visfatin levels and Disease Activity Score of 28 joints (DAS28) in RA patients.	813 RA patients 684 controls	Adiponectin levels were significantly higher in the RA group than in the control group. Circulating adiponectin levels were not associated with RA activity based on DAS28 and C-reactive protein (CRP) levels.
Liu (2020) [17]	Investigation whether AD exerts effect on Tfh cells in RA.	Human RA FLS Mice	AD-stimulated RA FLSs promote Tfh cell generation, which is mainly mediated by the secretion of soluble factor IL-6.
Zhang Y et al. (2020) [75]	Report determining the effect of baseline serum adiponectin levels in predicting the development of rheumatoid arthritis (RA).	3693 subjects with obesity followed-up for up to 29 years.	In this cohort of subjects with obesity, high serum adiponectin levels at baseline were associated with an increased risk of RA. Moreover, subjects with both high adiponectin and CRP levels at baseline were at particular risk of developing RA.

## Abbreviations

PG	prostaglandin
IL	interleukin
TGF-ß	transforming growth factor beta
TNF	tumor necrosis factor
IGF-1	insulin-like growth factor 1
MCP-1	monocyte chemoattractant protein-1
FLS	fibroblast-like synoviocyte
RASF	rheumatoid arthritis synovial fibroblast
RA FLS	rheumatoid arthritis synovial fibroblast
ADP	adiponectin
ACPA	anti-citrullinated protein antibodies
RF	rheumatoid factor
Tfh	follicular helper cells
PPAR	peroxisome proliferator-activated receptor
IRS	insulin receptor substrate
LMW	low molecular weight
MMW	medium molecular weight
HMW	high molecular weight
gAPN	globular adiponectin
fAPN	fibrous adiponectin
AdipoR	adiponectin receptor
PHH	primary human hepatocytes
CKD	chronic kidney disease
VEGF	vascular endothelial growth factor
MMP	matrix metalloproteinase
LPS	lipopolysaccharide
NO	nitric oxide
TRAP	tartrate-resistant acid phosphatase
Osx	osterix
